# Analysis of the impact of platinum-based combination chemotherapy in small cell cervical carcinoma: a multicenter retrospective study in Chinese patients 

**DOI:** 10.1186/1471-2407-14-140

**Published:** 2014-02-27

**Authors:** Long Huang, Ling-Min Liao, An-Wen Liu, Jian-Bing Wu, Xiao-Ling Cheng, Jia-Xin Lin, Min Zheng

**Affiliations:** 1 Department of Oncology, The Second Affiliated Hospital of Nanchang University, 1 Minde Road, Nanchang, Jiangxi, China; 2 Department of Ultrasound, The Second Affiliated Hospital of Nanchang University, Nanchang, China; 3 Department of Medical Imaging, Women And Children Health Institute Futian, Shenzhen, China; 4 Department of Gynecology, Cancer Center, Sun Yat-sen University, Guangzhou, China

**Keywords:** Cervical cancer, Small cell carcinoma, Platinum-based combination chemotherapy, Prognosis

## Abstract

**Background:**

Small cell cervical carcinoma (SCCC) is a rare, aggressive tumor with a poor prognosis. However, information in relation to its treatment is scarce due to the limited numbers of patients. The aim of this study was to establish whether platinum-based combination chemotherapy may by beneficial in this patient population.

**Methods:**

We carried out a multicenter, retrospective study comprising of 72 Chinese patients with SCCC. The patients were treated between 1995 and 2010 at Sun Yat-sen Memorial Hospital or the Cancer Center of Sun Yat-Sen University, and at the First Affiliated Hospital of Shantou University Medical College, China.

**Results:**

Of the 72 patients, 46/72 (63.9%) had Federation of Gynecology and Obstetrics (FIGO) stage Ia–Ib2 and 26/72 (36.1%) had stage IIa–IV disease. Surgery was performed in 63/72 (87.5%) patients, 61/72 (84.7%) patients received chemoradiotherapy and 35/72 (48.6%) received radiotherapy. The 3-year overall survival (OS) and disease-free survival (DFS) rates were as follows: Ia (100%, 100%); Ib1 (62%, 57%); Ib2 (53%, 48%); IIa (36%, 23%); IIb (29%, 21%); IIIb (50%, 50%); and IV (0%, 0%), respectively. The estimated 3-year OS and DFS rates in patients who received platinum-based combination chemotherapy (etoposide + cisplatin [EP], or paclitaxel + cisplatin [TP]) as part of their adjuvant treatment were 64.8% and 63.0%, respectively, compared to 25.2% and 22.0% in those who did not (P = 0.0003; P = 0.0003). Univariate analysis showed that platinum-based combination chemotherapy was associated with improved survival compared to other chemotherapy techniques or no chemotherapy (OS: HR = 0.227; 95% CI, 0.099–0.524; P = 0.001; DFS: HR = 0.210; 95% CI, 0.087–0.506; P = 0.001). Multivariate analysis identified FIGO stage, lymphatic metastasis and platinum-based combination chemotherapy as independent prognostic factors for improved survival in patients with SCCC.

**Conclusions:**

Platinum-based combination chemotherapy (with EP or TP) can improve the 3-year survival outcomes in patients with SCCC. Therefore, it should be considered an important component in a future standardized treatment strategy for SCCC.

## Background

Small cell cervical carcinoma (SCCC) was first described in 1957. It is a rare and aggressive cancer accounting for less than 3% of all cervical neoplasms [1-8]. Previous reports have shown that women diagnosed with SCCC have a higher frequency of lymph node metastases, lymphovascular invasion and recurrence, and have a poorer prognosis compared to women with other types of cervical malignancies [9-11]. The 5-year survival rates vary from 0% to 30% [12-14]. Moreover, SCCC is associated with rapid, distant metastasis to sites including the lung, liver, brain, bone, pancreas and lymph nodes, resulting in treatment failure in most cases [15-19].

The aggressive nature of SCCC and low survival rates mean that it is imperative to develop effective treatments to improve the outcomes of patients with SCCC. Due to its rarity, the time period required to enroll a sufficient number of patients for analysis is long, and an optimal, standardized treatment strategy for SCCC remains to be established. Most clinicians favor the use of platinum-based combination chemotherapy in the treatment of patients with SCCC because of its similarities to small cell lung cancer; however, its role in these therapies has not been clearly defined. In this study, we report a multicenter, retrospective trial comprising of 72 Chinese women diagnosed with SCCC, with the aim of defining an optimal platinum-based combination chemotherapeutic strategy for the treatment of patients with SCCC.

## Methods

### Patients

A total of 72 Chinese patients diagnosed with SCCC between January 1995 and January 2010 at the Cancer Center of Sun Yat-sen University (50 patients), the Sun Yat-sen Memorial Hospital of Sun Yat-Sen University (12 patients) and the First Affiliated Hospital of the Medical College of Shantou University (10 patients) were enrolled in this study. The selection criteria were as follows: confirmed histopathologic diagnosis of SCCC; no previous history of malignancy or a secondary primary tumor; detailed clinicopathologic data and follow-up data. Histologic classification of SCCC was performed by light microscopy, according to the definitions set by the World Health Organization (1981) for small cell cancer lung. Immunohistochemical neuron-specific enolase and silver staining had also been performed. The identification of any small cell component was considered sufficient for the patient to be included. The cancer was staged according to the International Federation of Gynecology and Obstetrics (FIGO) staging system. Clinical information for each patient was obtained from medical records. Disease status and vital data for each patient, including tumor recurrence and patient death, were obtained from a prospectively maintained hospital tumor registry. All patients agreed to participate in the study and gave written informed consent. This study was approved by the medical ethics committee of Second Affiliated Hospital of Nanchang University and Cancer Center of Sun Yat-Sen University.

### Treatment

The initial treatments consisted of primary surgery with or without neoadjuvant treatment, radiotherapy, or chemotherapy alone. With the exception of patients with surgical contraindications, all patients diagnosed with FIGO stage Ia–Ib2 SCCC underwent type II radical surgery, as described by Piver et al. [20]. Patients with stage IIa–IV disease received radical surgery, palliative surgery of no surgery (Table 1). Patients with high-risk factors underwent type II radical surgery followed by postoperative adjuvant therapy. All hysterectomy specimens were collected and subjected to pathological analysis. Individualized postoperative treatment consisted of radiotherapy, concurrent chemoradiotherapy or chemotherapy alone. Radiotherapy consisted of external pelvic irradiation administered using a multiportal technique, with a dose of 1.8–2.0 Gy administered daily to a total dose of 50 Gy over 5–6 weeks. The paraortic region was irradiated when metastases were detected in the common iliac or paraortic nodes, with a dose of 45 Gy administered over 5 weeks. Chemoradiotherapy was scheduled as follows: cisplatin (60 mg/m2) on day 1 and etoposide (100 mg/m2) daily for 3 days every 3 weeks for 4 cycles. The first 2 cycles of etoposide were given with concurrent radiotherapy on days 1 and 22. The subsequent two cycles of etoposide were given following radiotherapy. Chemotherapy alone was administered in the form of cisplatin (60–75 mg/m2) with either etoposide (100 mg/m2) or paclitaxel (135–175 mg/m2) over 3 hours on day 1, with cisplatin (60–75 mg/m2) on days 1 and 2. In this study, etoposide + cisplatin is defined as EP; paclitaxel + cisplatin is defined as TP. In general, 3–5 courses of chemotherapy were administered at 3-week intervals. The details of each patient’s treatment plan are given in Table 2.

**Table 1:** Treatment modalities for patients with SCCC according to FIGO stage

**Treatment**	**Number of patients (%)**	
	**FIGO stage Ia–Ib2**	**FIGO stage IIa–IV**
Surgery alone	3 (6.5)	3 (11.5)
Surgery + adjuvant chemotherapy	27 (58.7)	3 (11.5)
Surgery + adjuvant radiotherapy	1 (2.2)	4 (15.4)
Surgery + adjuvant concurrent chemoradiotherapy	13 (28.3)	9 (34.6)
Chemotherapy alone	1 (2.2)	0 (0)
Radiotherapy alone	0 (0)	4 (15.4)
Surgery + adjuvant concurrent chemoradiotherapy	0 (0)	2 (7.7)
No treatment	1 (2.2)	1 (3.8)

FIGO, International Federation of Gynecology and Obstetrics.

**Table 2:** Treatment plans and outcomes of the 72 cases of small cell cervical carcinoma

**Case**	**Age**	**FIGO stage**	**Treatment**			**Response to CT**	**Outcome (months)**
			**Surgery**	**PMCT**	**PMRT**		
1	29	Ia	Yes	EP ×4	No	CR	NED (98 M)
2	52	IIa	Yes	/	Yes	/	DOD (8 M)
3	27	Ib2	Yes	TP ×4	Yes	CR	NED (42 M)
4	58	Ib1	Yes	VAC ×4	Yes	PR	NED (23 M)
5	42	Ib2	Yes	VAC ×1 + EP ×6	No	PROG	DOD (17 M)
6	39	Ib2	Yes	CBP ×2 + TP ×4	No	SD	DOD (28 M)
7	54	IIIb	Yes	P ×1	No	PROG	DOD (25 M)
8	43	Ib1	Yes	/	No	/	NED (119 M)
9	36	Ib1	Yes	CAP ×2	No	PROG	DOD (22 M)
10	47	Ib1	Yes	CBP ×4	Yes	CR	NED (70 M)
11	46	Ib2	Yes	ITP ×4	Yes	SD	DOD (34 M)
12	38	IIa	Yes	CBP ×4	Yes	SD	DOD (38 M)
13	58	IIa	Yes	/	No	/	DOD (6 M)
14	48	IIb	Yes	/	Yes	/	DOD (2 M)
15	24	Ib2	Yes	IP ×1	Yes	PROG	DOD (5 M)
16	51	Ib1	Yes	VAC ×2 + EP ×2	No	PROG	DOD (23 M)
17	65	IIa	Yes	VAC ×2 + EP ×1	Yes	PROG	DOD (23 M)
18	37	IIb	No	/	Yes	/	DOD (6 M)
19	42	IIa	Yes	VAC ×2 + EP ×2	Yes	PR	DOD (33 M)
20	51	Ib2	Yes	EP ×4	No	CR	NED (43 M)
21	36	Ib1	Yes	TP ×4	Yes	PR	DOD (11 M)
22	48	Ib2	Yes	VAC ×2 + EP ×2	No	PR	NED (32 M)
23	55	Ib1	Yes	(IP + CP) ×5	No	PROG	DOD (28 M)
24	41	Ib1	Yes	EP ×3	No	PR	NED (20 M)
25	32	IIb	No	/	Yes	/	NED (63 M)
26	40	IIIb	Yes	TP ×5	Yes	PR	NED (64 M)
27	44	Ib1	Yes	EP ×4	/	CR	NED (59 M)
28	34	Ib1	No	VAC ×2 + EP ×2	No	PR	NED (22 M)
29	62	IIb	Yes	/	/	/	NED (20 M)
30	41	Ib2	Yes	EP ×4	Yes	CR	NED (17 M)
31	56	Ib1	Yes	EP ×4	Yes	CR	NED (46 M)
32	66	IIa	Yes	EP ×4	No	CR	NED (12 M)
33	39	IIb	No	/	Yes	/	DOD (12 M)
34	38	Ib1	Yes	EP ×4	Yes	SD	DOD (18 M)
35	54	Ib1	Yes	EP ×4	No	PR	NED (15 M)
36	39	Ib1	Yes	EP ×4	Yes	PR	NED (13 M)
37	38	Ib1	Yes	EP ×4	No	CR	NED (24 M)
38	51	IIa	Yes	EP ×4	No	CR	NED (25 M)
39	47	Ib1	Yes	EP ×4	Yes	PR	NED (26 M)
40	28	IV	No	/	/	/	DOD (1 M)
41	40	Ib2	No	/	/	/	NED (8 M)
42	48	Ib1	Yes	EP ×4	No	CR	NED (21 M)
43	57	IIa	Yes	EP ×4	Yes	CR	NED (11 M)
44	29	Ib2	Yes	/	No	CR	NED (29 M)
45	38	IIa	Yes	EP ×4	Yes	SD	DOD (18 M)
46	59	Ib1	Yes	EP ×4	No	/	NED (16 M)
47	61	Ib1	Yes	EP ×4	No	CR	NED (24 M)
48	43	Ib2	Yes	EP ×4	Yes	CR	NED (28 M)
49	40	Ib1	Yes	EP ×4	No	CR	NED (13 M)
50	42	Ib1	Yes	VAC ×4	No	CR	NED (74 M)
51	45	Ib1	Yes	/	No	/	DOD (19 M)
52	52	Ib2	Yes	EP ×4	No	CR	NED (46 M)
53	26	Ib1	Yes	EP ×4	/	CR	NED (32 M)
54	31	IIb	No	EP ×4 + VAC ×2	Yes	PR	DOD (17 M)
55	55	Ib1	Yes	EP ×4	No	CR	NED (50 M)
56	50	Ib1	Yes	EP ×5	Yes	PR	DOD (29 M)
57	44	Ib2	Yes	EP ×4	No	CR	DOD (33 M)
58	54	Ib1	Yes	/	Yes	/	DOD (38 M)
59	43	IIa	Yes	/	Yes	/	DOD (15 M)
60	40	Ib2	Yes	TP ×4	No	PR	DOD (34 M)
61	36	IIa	Yes	/	Yes	/	DOD (14 M)
62	46	Ib1	Yes	EP ×4	No	PR	DOD (49 M)
63	48	IIb	Yes	EP ×4	Yes	PR	DOD (20 M)
64	31	Ib1	Yes	EP ×4	/	CR	NED (10 M)
65	33	IIb	No	VAC ×2 + EP ×6	Yes	PROG	DOD (11 M)
66	67	IIa	Yes	EP ×4	Yes	CR	NED (18 M)
67	53	IIb	No	/	Yes	/	DOD (6 M)
68	38	IIa	Yes	EP ×4 + VAC ×2	Yes	PR	DOD (34 M)
69	42	Ia	Yes	EP ×4	No	PR	NED (32 M)
70	66	IIb	Yes	/	No	/	DOD (5 M)
71	45	Ib2	Yes	EP ×4	Yes	CR	NED (15 M)
72	41	Ib1	Yes	EP ×4	Yes	CR	NED (13 M)

PMCT, postoperative chemotherapy; PMRT, postoperative radiotherapy; CT, chemotherapy; EP, etoposide + cisplatin; VAC, vincristine + doxorubicin + cyclophosphamide; TP, paclitaxel + cisplatin; CBP, cyclophosphamide + bleomycin + carboplatin/cisplatin; CBP, cyclophosphamide + adriamycin + carboplatin/cisplatin; ITP, ifosfamide + pirarubicin + cisplatin; CR, complete response; PR, partial response; SD, stable disease; PROG, progressive disease; M, months; DOD, died of disease; NED, no evidence of disease.

### Statistical analysis

Overall survival (OS) and disease-free survival (DFS) were determined using Kaplan-Meier survival curves and the log-rank test. The Cox proportional hazards model was used to estimate the independent prognostic factors for OS and DFS. All analyses were performed using SPSS v.13.0 software (SPSS Inc.; Chicago, IL, USA). P-values <0.05 were considered statistically significant. The end points of all 72 patients were updated in May 2012.

## Results

### Clinicopathologic features of the 72 SCCC cases

**Figure 1:**
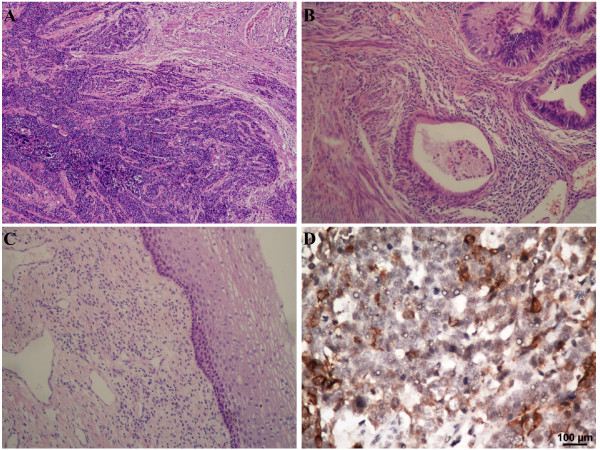
**Hematoxylin-eosin (H&E) stained SCCC tissue specimens. (A)** Small, round tumor cells with a high nuclear/cytoplasmic ratio are arranged in solid sheets with a diffuse or trabecular pattern (magnification, ×40). **(B)** Adenocarcinomatous components are present in some of the cells and clusters of small cell carcinoma cells can be seen in the vessels (magnification, ×40). **(C)** Small cell carcinoma cells pervade, but do not infiltrate, beneath the squamous epithelium (magnification, ×100). **(D)** Immunohistochemical staining for neuron-specific enolase shows positive cytoplasmic staining of the tumor cells (magnification, ×100).

The median age of the 72 patients enrolled in this study was 43 years (range: 24–66 years). Of these, 46 (63.9%) had FIGO stage Ia–Ib2 disease and 26 (36.1%) had stage IIa–IV disease. A mixed histologic pattern comprising of SCCC with squamous cell carcinoma or adenocarcinoma was diagnosed in 22 (30.6%) patients; the remaining 50 (69.4%) patients had a pure histologic type of SCCC. The 3-year OS rates were as follows: Ia (100%); Ib1 (62%); Ib2 (53%); IIa (36%); IIb (29%); IIIb (50%); and IV (0%). The 3-year DFS rates were as follows: Ia (100%); Ib1 (57%); Ib2 (48%0; IIa (23%); IIb (21%); IIIb (50%); and IV (0%). These rates were similar to those reported by Cohen et al. [21] based on an analysis of 52 patients diagnosed with small cell neuroendocrine carcinoma (SmCC) in Japan. They reported 4-year OS rates of Ib1 (63%); Ib2 (67%); IIb (30%); IIIb (29%); and IVb (25%), and 4-year DFS rates of Ib1 (59%); Ib2 (68%); IIb (13%); and IIIb (17%). The other clinicopathologic characteristics of the patients in our study are summarized in Table 3. Hematoxylin-eosin stained images of the specimens revealed small, round tumor cells arranged in solid sheets with scant cytoplasm, a high nuclear/cytoplasm ratio and indistinct cell borders (Figure 1A). A high proportion of mitotic cells were observed, and both squamous cell carcinoma and adenocarcinoma components were present, in addition to SCCC cells (Figure 1B). Vessel permeation was also observed in some specimens (Figure 1C). Many of the cases were positive for immunohistochemical neuron-specific enolase staining (Figure 1D).

**Table 3:** Univariate and multivariate Cox regression analysis of factors associated with overall survival (OS) and disease-free survival (DFS)

**Clinical variable**	**Overall survival**				**Disease free survival**			
	**Univariate analysis**		**Multivariate analysis**		**Univariate analysis**		**Multivariate analysis**	
	**Hazard ratio (95% CI)**	**P value**	**Hazard ratio (95% CI)**	**P value**	**Hazard ratio (95% CI)**	**P value**	**Hazard ratio (95% CI)**	**P value**
**Age(years)**	0.632 (0.318-1.255)	0.190			0.547 (0.276-1.085)	0.084		
≥40 (n = 27, 37.5%)								
<40 (n = 45, 62.5%)								
**Tumor homology**	1.717 (0.846-3.485)	0.135			1.681 (0.832-3.396)	0.148		
Mixed (n = 22, 30.6%)								
Pure (n = 50, 69.4%)								
**FIGO stage**	3.883 (1.936-7.790)	**<0.001**	3.478 (1.493-9.664)	**0.004**	3.572 (1.780-7.164)	**<0.001**	3.104 (1.377-7.978)	**0.007**
IIa–IV (n = 26, 36.1%)								
Ia–Ib2 (n = 46, 63.9%)								
**Tumor size**	2.057 (1.022-4.141)	**0.043**			2.320 (1.148-4.688)	**0.019**		
>4 cm (n = 27, 37.5%)								
≤4 cm (n = 45, 62.5%)								
**lymphatic metastasis**	3.235 (1.642-8.139)	**0.009**	3.617 (1.441-11.326)	**0.006**	4.237 (1.792-6.524)	**0.005**	4.852 (1.613-8.794)	**0.014**
Positive (n = 42, 58.3%)								
Negative (n = 30, 41.7%)								
**Vascular space invasion**	1.923 (0.967-3.824)	0.062			1.529 (0.764-3.059)	0.231		
Positive (n = 33, 45.8%)								
Negative (n = 39, 54.2%)								
**Depth of stromal invasion**	2.039 (0.908-4.577)	0.084			2.480 (0.1.107-5.559)	**0.027**		
>2/3 (n = 33, 45.8%)								
≤2/3 (n = 28, 38.9%)								
**Chemotherapy**	0.227 (0.099-0.524)	**0.001**	0.264 (0.099-0.671)	**0.003**	0.223 (0.101-0.496)	**<0.001**	0.221 (0.091-0.603)	**0.006**
EP or TP (n = 36, 50.0%)								
Others (n = 36, 50.0%)								

FIGO, International Federation of Gynecology and Obstetrics; CI, confidence interval; EP, etoposide + cisplatin; TP, paelitaxel + cisplatin; **Bold** indicates significant values.

### Relationship between different treatments and survival in patients with SCCC

**Figure 2:**
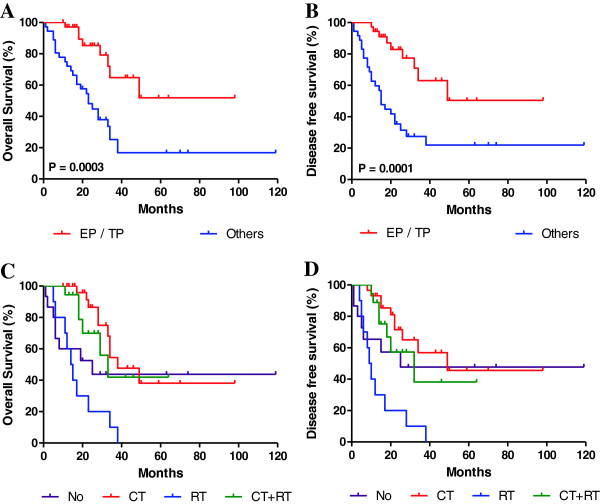
**Kaplan-Meier curves of overall survival (OS) and disease-free survival (DFS) for different treatment strategies in SCCC. (A and B)**: Comparisons between survival rates in patients who received adjuvant chemotherapy (etoposide + cisplatin [EP]; or paclitaxel + cisplatin [TP]) as part of their adjuvant treatment, compared to those who received alternative adjuvant therapies. **(C and D)** Comparisons between the survival rates in patients who received adjuvant chemotherapy (CT), adjuvant radiotherapy (RT), adjuvant chemoradiotherapy (CT + RT), or no adjuvant therapy (NO).

To determine whether platinum-based combination chemotherapy may be beneficial for the prognosis of patients with SCCC, the survival rates in patients who received adjuvant chemotherapy (EP or TP) were compared to those who received alternative adjuvant treatments. Our results revealed that the estimated 3-year OS rates were 64.8% vs. 25.2%, between these two groups, respectively (P = 0.0003; Figure 2A). The corresponding 3-year DFS rates were 63.0% vs. 22.0%, respectively (P = 0.0001; Figure 2B). Of the 72 patients enrolled in this study, 63 (87.5%) had undergone radical hysterectomy as the main mode of treatment. The treatment modalities for each patient are summarized in Table 2. We also assessed whether multimodal therapy improved prognosis. Due to the limited number of patients, we divided them into the following four groups: those who received adjuvant chemotherapy (n = 31), those who received adjuvant radiotherapy (n = 10), those who received adjuvant chemoradiotherapy (n = 18), and those who received no adjuvant therapy (n = 13). Patients who received adjuvant chemotherapy tended to have improved survival, with a 3-year OS rate of 54.5% compared to 0% in those who received adjuvant radiotherapy, 41.1% in those who received adjuvant chemoradiotherapy, and 43.8% in those who received no adjuvant therapy. Contrary to our expectations, patients who received adjuvant radiotherapy tended to have a poorer prognosis than those who received no radiation (Figure 2C,D). Based on these results, platinum-based combination chemotherapy should be recommended in the adjuvant treatment of patients with SCCC.

### Univariate and multivariate analyses to determine the prognostic factors in SCCC

To identify the prognostic factors for survival in patients with SCCC, we performed univariate and multivariate analyses of OS and DFS rates using the Cox proportional hazard model. Univariate analyses showed that FIGO stage (P < 0.001), lymphatic metastasis (P = 0.006), and EP or TP platinum-based combination chemotherapy (P = 0.001) were associated with the prognosis of patients with SCCC. Although vascular space invasion and depth of stromal invasion indicated poor survival, the effects were not statistically significant. In contrast, age, tumor size and tumor homology were not associated with prognosis in SCCC (P > 0.05).

After adjusting for potential confounding factors, multivariate analyses identified FIGO stage (HR, 3.478; 95% CI, 1.493–9.664; P = 0.004), lymphatic metastasis (HR, 3.617; 95% CI, 1.441–11.326; P = 0.006), and EP or TP platinum-based combination chemotherapy (HR, 0.264; 95% CI, 0.099–0.671; P = 0.003) as significant independent prognostic factors for overall survival in SCC (Table 2).

## Discussion

SCCC is a rare malignancy. Consequently, prospective randomized trials to assess the impact of various treatments on patient outcome have not been possible. Attempts by the Gynecologic Oncology Group to study SCCC have failed due to insufficient patient numbers. In recent years, novel methods for the treatment of SCCC have attempted to replicate successful treatments for small cell lung carcinoma. The management of SCCC has also been influenced by the success of concurrent chemoradiotherapies in other types of locally advanced cervical cancer. Despite these approaches, an optimal treatment strategy for SCCC remains to be established. In this study, we carried out a retrospective, multicenter trial consisting of 72 Chinese patients with SCCC in order to determine the most effective platinum-based combination chemotherapeutic strategy to improve survival outcome in these patients.

Currently, surgical resection only plays a limited role in patients with stage I–II small cell lung carcinoma [22], and its role in stage III disease is considered investigational [23,24]. In contrast, there have been few studies on the role of surgery in the treatment of SCCC, and it is unclear which patients, if any, should undergo radical hysterectomy as opposed to primary treatment with combined chemotherapy and radiotherapy. However, most gynecologic oncologists and patients in China favor radical hysterectomy. The majority of patients with SCCC enrolled in this study (87.5%) had undergone radical hysterectomy as the main mode of treatment. Although several reports have suggested that radical hysterectomy does not prolong patient survival in SCCC [25], our results have indicated that it was an important component in the multimodal treatment of SCCC. In our centers, the standard approach to treating patients with SCCC is radical hysterectomy followed by several cycles of platinum-based combination chemotherapy.

Platinum-based combination chemotherapy has been reported to be as beneficial in patients with SCCC as it is for patients with lung cancer [26]. However, SCCC differs from small cell lung cancer due to its lower risk of brain metastasis and this benefit remains to be confirmed. Several case studies and small-series reports have indicated that platinum-based combination chemotherapy may have positive outcomes in patients with SCCC: Hoskins et al. reported a 3-year failure-free rate of 80% in 31 patients with early stage disease (stage I–II) who underwent combination therapies that included etoposide and cisplatin [25]; Gardner et al. reported that etoposide/platinum-based chemotherapy was effective for neuroendocrine carcinomas of the gynecologic tract but not for well-differentiated carcinoid tumors [27]; and Chang et al. found that chemotherapies containing a combination of cisplatin and etoposide could be effective in patients with early stage SCCC following radical hysterectomy [18]. Although comparisons between series can be problematic due to selection bias and different treatment modalities, our data supports the role of platinum-based combination chemotherapy in the treatment of patients with SCCC. Patients who received chemotherapy (with EP or TP) as part of their adjuvant treatment had estimated 3-year OS and DFS rates of 64.8% and 63.0%, respectively, compared to 25.2% and 22.0%, respectively, in those who received alternative adjuvant therapies (P < 0.05).

Several studies have reported that disease stage was the strongest predictor of outcome, and that other factors, including age, tumor size, depth of stromal invasion, vascular space invasion, were also prognostic factors in SCCC [28,29]. However, our univariate and multivariate analyses found no association between age, depth of stromal invasion or vascular space invasion with patient outcome in SCCC. In contrast, we found that FIGO stage, lymphatic metastasis and platinum-based combination chemotherapy were independent prognostic factors for improved survival in SCCC. To our knowledge, this is the first report to reveal that platinum-based combination chemotherapy is an independent prognostic factor for patients with SCCC.

## Conclusions

Our study has demonstrated that improved survival outcomes can be achieved in patients with SCCC by incorporating platinum-based combination chemotherapy into adjuvant treatment strategies. Although this study was retrospective in design, it is one of the largest series reported to date, and the results could be an important contribution to our understanding and future therapies of this rare and aggressive tumor.

## Competing interests

The authors declare that they have no competing interests.

## Authors’ contributions

LH and LML conceived the study, was responsible for its design and coordination, participated in the analysis and interpretation of the data, as well as in drafting and revising all versions of the manuscript. AWL and JBW participated in the study design and revising the manuscript. XLC and JXL participated in the study design and data collection. MZ participated in the study design and critical revision of the manuscript. All authors read and approved the final manuscript.

## Pre-publication history

The pre-publication history for this paper can be accessed here:

http://www.biomedcentral.com/1471-2407/14/140/prepub
